# Socioeconomic status of the elderly MS population compared to the general population: a nationwide Danish matched cross-sectional study

**DOI:** 10.3389/fneur.2023.1214897

**Published:** 2023-06-13

**Authors:** Malthe Faurschou Wandall-Holm, Rolf Pringler Holm, Luigi Pontieri, Finn Sellebjerg, Melinda Magyari

**Affiliations:** ^1^Danish Multiple Sclerosis Registry, Department of Neurology, Copenhagen University Hospital – Rigshospitalet, Glostrup, Denmark; ^2^Department of Neurology, Danish Multiple Sclerosis Center, Copenhagen University Hospital – Rigshospitalet, Glostrup, Denmark

**Keywords:** multiple sclerosis (MS), aging, income, education, socioeconomics, family, patient—centered care

## Abstract

**Introduction/objectives:**

Multiple sclerosis (MS) leads to physical and cognitive disability, which in turn impacts the socioeconomic status of the individual. The altered socioeconomic trajectory combined with the critical role of aging in MS progression could potentially lead to pronounced differences between MS patients and the general population. Few nations have the ability to connect long-term clinical and socioeconomic data at the individual level, and Denmark's robust population-based registries offer unique insights. This study aimed to examine the socioeconomic aspects of elderly Danish MS patients in comparison to matched controls from the general population.

**Methods:**

A nationwide population-based study in Denmark was conducted, comprising all living MS patients aged 50 years or older as of 1 January 2021. Patients were matched 1:10 based on sex, age, ethnicity, and residence with a 25% sample of the total Danish population. Demographic and clinical information was sourced from the Danish Multiple Sclerosis Registry, while socioeconomic data were derived from national population-based registries containing details on education, employment, social services, and household characteristics. Univariate comparisons between MS patients and matched controls were then carried out.

**Results:**

The study included 8,215 MS patients and 82,150 matched individuals, with a mean age of 63.4 years (SD: 8.9) and a 2:1 female-to-male ratio. For those aged 50–64 years, MS patients demonstrated lower educational attainment (high education: 28.3 vs. 34.4%, *P* < 0.001) and fewer received income from employment (46.0 vs. 78.9%, *P* < 0.001), and working individuals had a lower annual income (48,500 vs. 53,500€, *P* < 0.001) in comparison to the controls. Additionally, MS patients within this age group were more likely to receive publicly funded practical assistance (14.3 vs. 1.6%, *P* < 0.001) and personal care (10.5 vs. 0.8%, *P* < 0.001). Across the entire population, MS patients were more likely to live alone (38.7 vs. 33.8%, *P* < 0.001) and less likely to have one or more children (84.2 vs. 87.0%, *P* < 0.001).

**Conclusion:**

MS presents significant socioeconomic challenges among the elderly population, such as unemployment, reduced income, and increased dependence on social care. These findings underscore the pervasive impact of MS on an individual's life course, extending beyond the clinical symptoms of cognitive and physical impairment.

## 1. Introduction

Over the last few decades, the mean age among patients with multiple sclerosis (MS) has increased (1, 2). This trend may be attributed to advances in diagnostics, the impact of disease-modifying therapies (DMT), improved supportive care enhancing prognosis, and a general increase in life expectancy in the general population. Another contributing factor is the rise in the incidence of late-onset MS, which includes individuals experiencing their first clinical symptom after the age of 50 (1, 2). This demographic shift is important as MS is primarily considered a disease of young adults. The approach to elderly patients is likely to be markedly different due to unique clinical characteristics, comorbidities, and daily life needs within this select patient group.

Traditional clinical parameters are limited in providing a comprehensive view of an MS population, as functional scores such as the Expanded Disability Status Scale (EDSS) have several shortcomings (3, 4). Consequently, socioeconomic factors become crucial in describing the state of patients with MS. These parameters directly impact patient's lives and are often more relatable for both patients and decision-makers, particularly when considering the long-term consequences of MS (5).

Universally, the general population's need for personal care and practical assistance increases with age (6). However, in a disease like MS, where disability accumulates over time, the interaction with age may further amplify this effect. It is thus essential to investigate the growing population of older patients with MS, especially given the high societal cost of caretaking for these patients (7, 8).

An MS diagnosis has also been shown to affect personal finances adversely. Patients with MS tend to experience reduced workability and the proportion of patients receiving disability pension or lacking labor-related income increases following an MS diagnosis (9–11). Our previous research has demonstrated that Danish patients with MS are at a higher risk of losing all income from earnings and face a much higher likelihood of receiving disability pension than healthy controls (12).

This study aims to describe multiple aspects of the aging population with MS, focusing on differences in employment, income, workability, and family-related outcomes in patients with MS over 50 years old by comparing them to matched individuals from the general population.

## 2. Methods

### 2.1. Study design and study population

We conducted a matched nationwide cross-sectional study in Denmark, with a reference date of 1 January 2021. From the Danish Multiple Sclerosis Registry (13) (DMSR), we identified all patients with a diagnosis of definite MS. To be included in the study, patients had to be above 50 years of age, alive, and living in Denmark at the reference date. We matched each patient to 10 controls from a 25% random sample of the entire Danish background population (excluding patients with MS) based on sex, exact age, ethnicity, and geographic region at the reference date. To investigate parental status, we constructed a population that included all children born to individuals from the MS population and the 25% random sample, selecting the children of all individuals enrolled in the study.

In addition, we performed a longitudinal sub-analysis on work-related measures that included all participants from the cross-sectional study aged between 50 and 64 years from 1980 to 2020. The cutoff at 64 years was due to the Danish state pension age of 65 years for people born before 1 July 1959. Since then, the state pension age has gradually increased to adjust for increased life expectancy and demographical changes, but the threshold at 64 years was set to reduce temporal selection bias. We collected their annual economic data for each integer age level within the specified age range. Subsequently, we created graphical representations, displaying the proportion of individuals receiving disability pension or having no income from employment and the annual income in euros for those with an income from work and were not receiving disability pension to analyze trends across age levels.

### 2.2. Data sources and variables

The unique personal identification code assigned to all Danish citizens or individuals with a permanent address residing in the country for more than 3 months enabled cross-linkage between registries on the individual level (14).

#### 2.2.1. Clinical

The DMSR is a nationwide, population-based registry containing information on all patients with MS since 1948. Currently, data are obtained from each of the 13 MS clinics distributed around the country and are entered directly into an online platform by clinicians. Since the introduction of DMTs on the Danish market in 1996, data entry on treated patients has been mandatory. The data are the basis for national clinical quality indicators ensuring a high degree of completeness and validity (15). The DMSR contains clinical data on demographic information, diagnostics, disease status, imaging, and more.

From the DMSR, we collected age, sex, age at clinical onset, current phenotype, latest EDSS score within 2 years, relapse activity, and time since the last clinical visit. We calculated disease duration as the difference in years between the onset and the reference date.

#### 2.2.2. Socioeconomic

Socioeconomic and demographic information was collected from several national Danish population-based registries such as the Population Statistics Register (PSR), the Income Statistics Register (ISR), the Employment Classification Module (AKM), the Sickness Benefits Statistics Register (SBSR), the Danish Education Register (DER), the Elderly Documentation Register (EDR), the Immigrants and Descendants Register (IDR), the Danish Rational Economic Agents Model (DREAM), the Social Pensions Register (SOCP), the Cause of Death Register (CDR), the Historical Migration Register (VNDS), and the Register-based Labor Force Statistics (RAS). Through the Fertility Database (FER) we identified children of study participants from both the MS and general population. The nationwide registers have an expected coverage of 97% of the population and a high level of validity (16). For the cross-sectional study, all data were collected for 2020 except for work absence from SBSR, which was only available for 2019.

In Denmark, the municipality office functions as a local government authority. It provides a range of social services, including assistance with personal care (such as dressing, bathing, and toileting) and practical help (such as cleaning, grocery shopping, and laundry). The municipality conducts an individual assessment to determine the required level of support. Information about the services provided is reported to the EDR, from which we collected data on the weekly amount of personal and practical help.

From the AKM, we collected information about the occupational classification based on the International Standard Classification of Occupations (ISCO) to determine the primary source of income grouped as either “wage earner,” “pensioner,” “long-term unemployed,” or “others.” Long-term unemployed individuals are either unemployed for more than half a year or recipients of social security benefits, which is financial assistance provided to individuals who are unemployed or have a low income.

From the PSR, we collected cohabitation and marital status. Cohabitation was defined as living with another adult (18 years or older) or living alone.

We collected gross annual income from primary and secondary employment and benefits or allowances from the ISR. The income was subsequently adjusted using the net price index with 2015 as the reference year to account for inflation and allow for direct purchasing power comparisons.

From RAS, we collected information on working hours categorized as either “full-time” or “part-time.” An individual was considered to be working full-time if they, on average, worked more than or equal to 32 h a week annually and part-time if they worked fewer than 32 h a week.

From SBSR, we collected information on the duration of long-term absence from work due to illness, with long-term defined as having 30 or more days of absence. In Denmark, an employer can receive public financial aid if an employee has 30 or more days of absence due to illness. As such, employers are highly incentivized to report long-term illness to the municipality office, but this registry does not ensure complete coverage.

From the DER, we collected the highest, completed education and converted it into three categories according to the International Standard Classification of Education (ISCED) classification (17): ISCED level 0–2, ISCED level 3–4, and ISCED level 5–8, corresponding to low, medium, and high educational levels. For an extended description of the ISCED classification and the translation from Danish to English terminology, see Supplementary Description 1.

From DREAM, we collected data on whether an individual had received a social transfer payment designated as “disability pension.” In Denmark, disability pension is public support benefit provided to individuals whose work capacity is permanently and substantially reduced, rendering them unable to support themselves in the labor market. To apply for disability pension, a formal application must be submitted to the municipal office. The assessment process for eligibility involves an evaluation of the individual's work ability and potential for reskilling or receiving additional support for employment. A medical evaluation conducted by a healthcare professional is typically part of this process.

The structure of the disability pension system underwent a reform in 2003 to simplify and restrict access. Prior to 2003, the benefit was distributed across four levels, depending on the age of the individual and the degree of loss of working ability for individuals of working age (18–65 years). After 2003, the benefit was reduced to one level (adjusted for cohabitation) and was primarily granted to individuals between 40 and 65 years of age although exceptions for individuals under 40 years are permitted. In our study, we categorize the status of “receiving disability pension” binarily irrespective of whether it was granted before or after the 2003 reform.

### 2.3. Statistical analysis

Patient and control characteristics are displayed as frequencies with corresponding percentages, mean values, and standard deviation (SD) or median values with the 1st and 3rd quartiles. In the longitudinal analysis, we display the proportion of persons with disability pension and no taxable income (not mutually exclusive) and median annual income in euros with whiskers displaying the first and third quartile for those receiving a taxable income.

For the cross-sectional descriptive analysis, missing data are included as a missing category. For the longitudinal analysis, there was no missing data on disability pension. In the rare case of a patient missing a record for one or more income years in ISR (present in 0.56% of patients), the years with missing information were disregarded.

For significance testing of differences between groups, multiple models were applied according to the outcome variable and accounting for the clustering of matches. For nominal outcomes, we used a Rao-Scott chi-square test. For all other outcomes, we used a generalized estimating equation: binary outcomes had a logit link function and ordinal outcomes had a multinomial distribution with a cumulative logit link, and for non-normally distributed continuous outcomes, we used a Wald-type rank test (18). *P*-values were adjusted for multiplicity by the Benjamini and Hochberg (FDR) procedure.

No sensitivity analyses were performed. Data management and table creation were carried out using SAS version 9.4 (SAS Institute Inc., Cary, NC, USA) and figures were created using R version 3.4.3.

### 2.4. Ethics, approvals, and data access

Informed consent or ethical approval is not a requirement for anonymized register-based studies in Denmark. The study is conducted under the Danish GDPR and registered at the Knowledge Center for Data Reviews, the data-responsible entity of the Capital Region of Denmark, approved by the Danish Data Protection Agency. Access to data is available upon qualified request.

All cells containing information from fewer than five subjects (or neighbors allowing cross-cell calculations) are censored to avoid personally identifiable data. Data preparation was performed on secure servers hosted by Statistics Denmark.

## 3. Results

**Figure 1:**
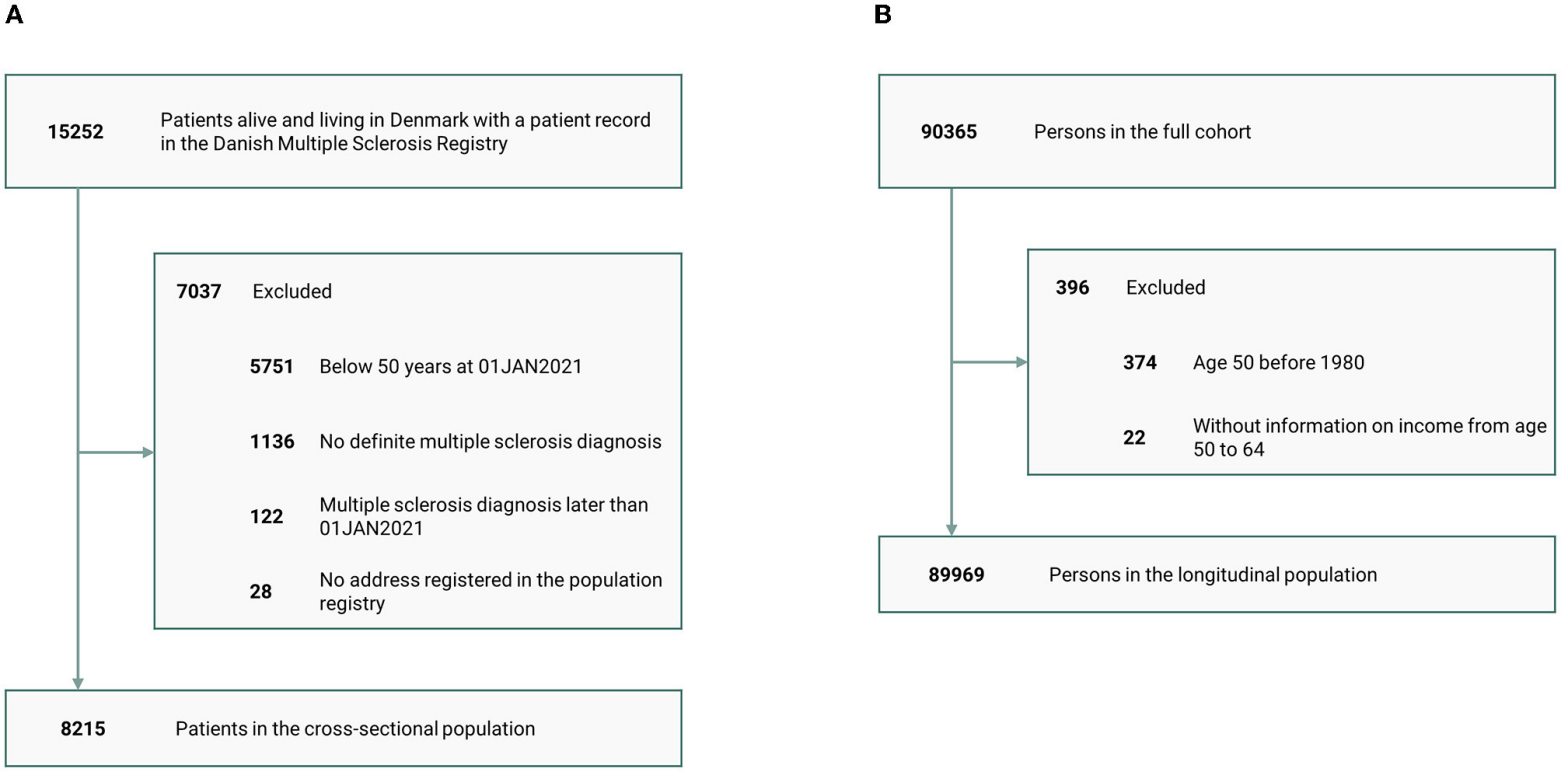
Patient disposition. **(A)** Cross-sectional study and **(B)** longitudinal study.

A total of 15,252 patients in the DMSR were screened for eligibility, and 8,215 met the inclusion criteria and were enrolled in the study population (Figure 1A). Each patient was matched with 10 individuals, resulting in a control group of 82,150 individuals from the background population. The mean age of the entire population was 63.4 years (SD: 8.9) at the reference date with a female-to-male ratio of 2:1 (68.3% were female participants), and 99.2% were of Danish ethnicity. For patients who had an EDSS score recorded within the last 2 years, the median score was 3.5 (Q_1_-Q_3_= 2.0–6.0); however, 44% (*n* = 3,585) did not have a recent EDSS assessment. See Table 1 for further characteristics of the MS population.

**Table 1:** Demographic and clinical characteristics of the MS population.

	**MS population**
Number of patients	8,215
Age, years, mean (SD)	63.4 (8.9)
Age at onset, years, mean (SD)	39.3 (11.2)
Disease duration, years, mean (SD)	24.1 (12.6)
Latest EDSS within 2 years, median (Q1–Q3), *n*_miss_	3.5 (2.0–6.0), *n*_miss_ = 3,585
Time since last visit, years, median (Q1–Q3), *n*_miss_	1.3 (0.4–11.5), *n*_miss_ = 0
**Sex**, ***n*** **(%)**	
Male	2,602 (31.7)
Female	5,613 (68.3)
**Ethnicity**, ***n*** **(%)**	
Danish	8,147 (99.2)
Other	68 (0.8)
**Phenotype**, ***n*** **(%)**	
RRMS	3,664 (44.6)
SPMS	2,043 (24.9)
PPMS	1,364 (16.6)
Unspecified	1,144 (13.9)
**One or more relapses recorded in the last 2 years**, ***n*** **(%)**	
Yes	449 (5.5)
No	7,766 (94.5)

EDSS, expanded disability status scale; RRMS, relapsing-remitting multiple sclerosis; SPMS, secondary progressive multiple sclerosis; PPMS, primary-progressive multiple sclerosis.

Table 2 presents the educational- and labor-related parameters. For individuals aged 65 years or older (mean age of 72.2 years), the difference in the educational level between people with MS and the controls was minor and not statistically significant. However, for patients younger than 65 years (mean age of 56.9 years), a greater proportion of individuals with MS had a low educational level (22.5 vs. 19.6%) and a medium educational level (48.8 vs. 44.9%), while a smaller proportion had a high educational level (28.3 vs. 34.4%). Overall, the education levels of both people with MS and the controls increased over time when comparing the two age groups.

**Table 2:** Socioeconomic parameters in the MS and the matched population.

	<**65 years**			≥**65 years**		
	**Background**	**MS**	***P*** **-value**	**Background**	**MS**	***P*** **-value**
Number of persons	50,040	5,004	–	32,110	3,211	–
Age, mean (SD)^a^	56.9 (4.2)	56.9 (4.2)	–	72.2 (5.7)	72.2 (5.7)	–
**Educational level**^b^, ***n*** **(%)**						
ISCED 0–2 (low)	9,804 (19.6)	1,128 (22.5)	<0.001	10,074 (31.4)	1,048 (32.6)	0.08
ISCED 3–4 (medium)	22,473 (44.9)	2,443 (48.8)		13,285 (41.4)	1,345 (41.9)	
ISCED 5+ (high)	17,233 (34.4)	1,414 (28.3)		8,470 (26.4)	808 (25.2)	
Missing	530 (1.1)	19 (0.4)		281 (0.9)	10 (0.3)	
**Primary source of income**, ***n*** **(%)**						
Wage earner	38,311 (76.7)	1,987 (39.7)	<0.001	3,309 (10.3)	135 (4.2)	<0.001
Pensioner^c^	6,280 (12.5)	2,400 (48.0)		28,597 (89.1)	3,060 (95.3)	
Long-term unemployment	3,737 (7.5)	541 (10.8)		127 (0.4)	10 (0.3)	
Other	1,712 (3.4)	76 (1.5)		77 (0.2)	6 (0.2)	
Receiving income from work^d^, *n* (%)	39,495 (78.9)	2,301 (46.0)	<0.001	5,008 (15.6)	189 (5.9)	<0.001
**If receiving income from work** ^d^						
Number of persons	39,495	2,301		5,008	189	
Annual income in €, median (Q1–Q3)	53,500 (41,000–68,000)	48,500 (25,000–63,500)	<0.001	20,000 (3,000–49,500)	15,500 (2,000–45,500)	<0.001
**Full time or part-time**, ***n*** **(%)**						
Full time	28,286 (71.6)	1,032 (44.9)	<0.001	1,379 (27.5)	37 (19.6)	0.05
Part time	7,352 (18.6)	1,051 (45.7)		1,853 (37.0)	60 (31.7)	
Missing	3,856 (9.8)	218 (9.5)		1,776 (35.5)	92 (48.7)	
More than 30 days of absence	3,438 (8.7)	309 (13.4)	<0.001	219 (4.4)	8 (4.2)	0.93

^a^Matching variable.

^b^The highest completed education converted into three categories according to the International Standard Classification of Education (ISCED) classification: ISCED level 0–2, ISCED level 3–4, and ISCED level 5–8, corresponding to low, medium, and high educational levels.

^c^For the age group <65 years, this is mainly disability pensions (>95%), but also a few other minor early retirement pension schemes.

^d^Additionally not receiving disability pension.

Table 2 also highlights a significant difference in the proportion of people receiving income from work regardless of age. Among those under 65 years old, only 46.0% of people with MS received income from employment compared to 78.9% among controls. For individuals aged 65 years or older, the proportions were 5.9% for those with MS vs. 15.6% for controls. The primary source of income exhibited a similar pattern: among individuals under 65 years of age, only 39.7% of people with MS had “labor” as their primary source of income compared to 76.6% among controls. Conversely, 48.0% of the people with MS had “pension” as their primary source of income compared to 12.5% among controls. The overall trend persists among those aged 65 years or older though the differences were less pronounced.

**Figure 2:**
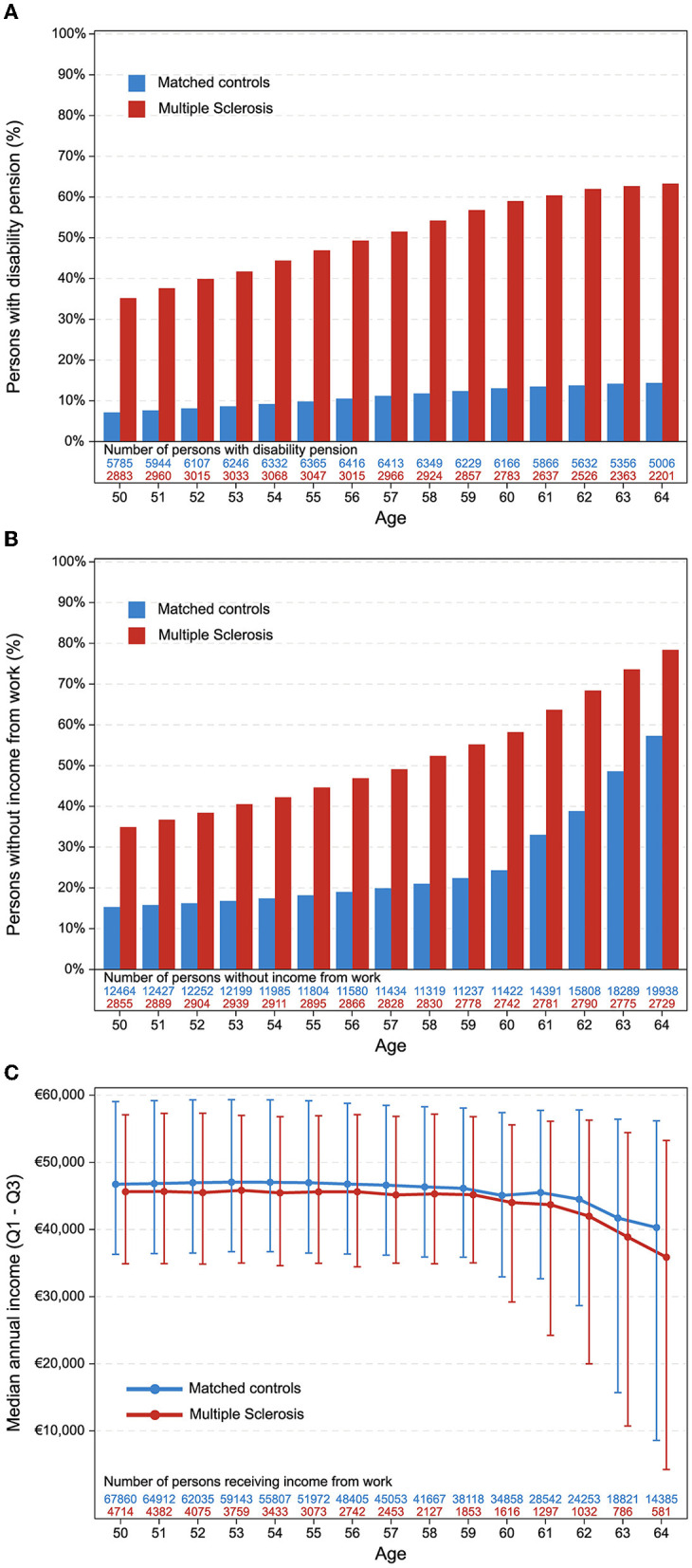
Longitudinal analysis. All panels display variable distributions according to age and group with “number of persons” at the bottom indicating the number of individuals in each data point. **(A)** Persons with disability pension. **(B)** Persons without income from work. Individuals can be present in both **(A, B)**. **(C)** Median annual income for persons with an income. Individuals can only contribute data to **(C)**, if not present in **(A)** or **(B)** (i.e., do not have disability pension and do have income from work).

Figure 2 presents results from the longitudinal analysis, allowing individuals to contribute data to each integer age (Patient disposition, Figure 1B). Figure 2A displays the prevalence of disability pension recipients among individuals aged 50–64 years with and without MS, illustrating a consistent relative difference throughout this senior working age. Figure 2B depicts the varying prevalence of individuals with no income from employment in the same age range for those with and without MS. This illustrates that among the control group, many individuals gradually stop receiving income from work without being granted disability pensions as they age. It is important to note that some individuals will be present in both figures, but not all.

Among individuals under 65 years of age who receive income from employment, we found a statistically significant difference in the median yearly income between those with MS and controls (48,500€ vs. 53,500€). Additionally, the proportion of people working part-time was more than double among people with MS (45.7 vs. 18.6%), and the percentage of people who had more than 30 sick days per year was also higher among the MS population (13.4 vs. 8.7%). Figure 2C illustrates the distinct median yearly incomes for individuals aged 50–64 years who have an income but are not on disability pension, displaying that the median income is slightly lower among people with MS across all age groups. However, if an individual with MS maintains income from employment, they follow a similar trajectory as the controls.

Table 3 presents information on municipal social services provided. Overall, the proportion of individuals receiving practical help or personal care increased with age when comparing those younger than 65 years to those older. In the under-65 age group, people with MS received practical help almost nine times more frequently than those without MS (14.3 vs. 1.6%). However, the amount of practical help received was similar (2.0 vs. 1.7 monthly hours). In the same age group, 10.5% of people with MS received personal care with a median of 18.3 monthly hours compared to just 0.8% of people without MS who received personal care with a median of 4.7 monthly hours. This trend persisted among individuals aged 65 years and older.

**Table 3:** Municipal services provided.

**Population**	<**65 years**			**65 years or more**		
	**Background**	**MS**	***P*** **-value**	**Background**	**MS**	***P*** **-value**
Number of persons	50,040	5,004	–	32,100	3,210	–
Practical help, *n* (%)	777 (1.6)	715 (14.3)	<0.001	2,361 (7.4)	909 (28.3)	<0.001
If yes, hours, median (Q1–Q3)	1.7 (0.8–2.4)	2.0 (1.3–3.8)	<0.001	1.7 (0.9–2.6)	2.1 (1.3–4.2)	<0.001
Personal care, *n* (%)	376 (0.8)	524 (10.5)	<0.001	1,552 (4.8)	866 (27.0)	<0.001
If yes, hours, median (Q1–Q3)	4.7 (1.2–13.7)	18.3 (4.3–52.0)	<0.001	3.7 (1.0–11.6)	21.4 (5.2–61.8)	<0.001

Table 4 presents family characteristics for the study population. People with MS tended to live alone more frequently (38.7 vs. 33.8%) and had a lower marriage rate (56.3 vs. 59.2%) compared to the control group at the reference date. Upon examining divorce prevalence, a difference between sexes emerged: male participants with MS had a higher proportion of divorce compared to male controls (18.9 vs. 15.8%), while this difference was not observed among female participants with MS (19.5 vs. 19.3%).

**Table 4:** Family characteristics.

	**Full population**			**Female**			**Male**		
	**Background**	**MS**	***P*** **-value**	**Background**	**MS**	***P*** **-value**	**Background**	**MS**	***P*** **-value**
Number of persons	82,150	8,215	–	56,130	5,613	–	26,020	2,602	–
**Cohabitation**, ***n*** **(%)**									
With another	54,368 (66.2)	5,034 (61.3)	<0.001	35,966 (64.1)	3,385 (60.3)	<0.001	18,402 (70.7)	1,649 (63.4)	<0.001
Alone	27,782 (33.8)	3,181 (38.7)		20,164 (35.9)	2,228 (39.7)		7,618 (29.3)	953 (36.6)	
**Marital status**, ***n*** **(%)**									
Married (+ separated)	48,655 (59.2)	4,629 (56.3)	<0.001	32,282 (57.5)	3,080 (54.9)	<0.001	16,373 (62.9)	1,549 (59.5)	<0.001
Divorced	14,951 (18.2)	1,589 (19.3)		10,843 (19.3)	1,096 (19.5)		4,108 (15.8)	493 (18.9)	
Unmarried	11,494 (14.0)	1,259 (15.3)		7,009 (12.5)	816 (14.5)		4,485 (17.2)	443 (17.0)	
Widow(er)	7,050 (8.6)	738 (9.0)		5,996 (10.7)	621 (11.1)		1,054 (4.1)	117 (4.5)	
**Has at least one child**, ***n*** **(%)**	71,502 (87.0)	6,913 (84.2)	<0.001	50,020 (89.1)	4,813 (85.7)	<0.001	21,482 (82.6)	2,100 (80.7)	0.02
If yes, number of children, mean (SD)	2.2 (0.9)	2.1 (0.9)	<0.001	2.2 (0.9)	2.1 (0.8)	<0.001	2.2 (0.9)	2.2 (0.9)	0.01
If yes, parent age at first child, mean (SD)	26.5 (5.3)	26.2 (5.0)	<0.001	25.6 (5.0)	25.4 (4.7)	<0.001	28.5 (5.4)	27.9 (5.3)	<0.001

The proportion of people having children was lower in the MS population at 84.2% compared to 87.0% among controls. This difference was even more pronounced among female participants (85.7 vs. 89.1%) but less among male participants (80.7 vs. 82.6%). Among those with at least one child, the average number of children was nearly identical (2.1 vs. 2.2), and the parent's age at the birth of the first child was also similar (26.2 vs. 26.5 years), with male participants generally being older than female participants (27.9–28.5 vs. 25.4–25.6 years).

## 4. Discussion

In this Danish nationwide population-based study, we found significant socioeconomic differences between people with MS and the matched general population, such as reduced employment, lower earnings, and a higher reliance on social benefits. Various socioeconomic factors can serve as indicators of an individual's functional level, and we focused on education, employment, income, and family-related factors. Investigating socioeconomic outcomes is essential as MS typically has onset in early life, affecting individuals functionally and financially for the majority of their lives. Moreover, MS imposes considerable direct and indirect costs on society, especially evident in the growing proportion of elderly individuals in the MS population (19, 20).

In individuals younger than 65 years, the highest achieved educational level was lower in the MS population compared to the matched controls from the background population. However, for those aged 65 years or older, no difference in the educational level was observed between the two groups. Generally, both the MS and control population had lower education levels among those aged 65 years or older compared to those under 65 years. This finding implies that people with MS may have benefited less from the overall increase in educational levels observed in recent decades. A potential explanation for the observed divergence in educational levels between the populations above and below 65 years of age could be the increased cognitive demands associated with higher education levels. As a result, the cognitive impairment and fatigue commonly experienced by MS patients might hinder them from maintaining pace. Nevertheless, previous studies from other countries have reported mixed findings on this subject. Some found no differences in the educational level between the MS population and the background population (21), while other studies reported a higher educational level among people with MS (22, 23). The observed variations might result from important differences in data sources and study designs: discrepancies could arise from different data structures and classification of socioeconomic indicators (such as grouping of educational levels). Additionally, one of the studies only matched on age and sex and did not account for reported differences in ethnicity or geographical factors (22). The other two studies did not consider sex, age, ethnicity, or geographical differences when comparing the MS population, which had a highly specific composition of characteristics, to more general populations (21, 23). Furthermore, when conducting inter-country comparisons, it is crucial to consider differences in access and funding for education up to the university level. In Denmark, education is provided free of charge, and all residents are entitled to student grants. Moreover, if a person is disabled, the state offers additional financial and social support.

People with MS demonstrated a weaker connection to the labor market with a significantly lower proportion receiving income from employment and a lower proportion employed full-time. Additionally, a higher percentage of people with MS had over 30 days of absence or received disability pension. Among those receiving income from employment, people with MS had lower annual earnings in 2020. However, when examining temporal trends, the difference in earnings showed considerable year-to-year fluctuations (Supplementary Figure 1). Numerous studies have investigated employment-related outcomes, revealing a wide range of differences due to variations in data sources, study sizes, and social systems across countries. A study conducted in New Zealand demonstrated a significant disparity between the MS population and the general population (23), and a Danish study from 2010 found that among those receiving disability pension, the median time to obtain it was 10 years for MS patients and 24 years for controls (11). It is important to note that the eligibility criteria for disability pension are subject to temporal variations in accordance with contemporary implementations of social policies. All previous studies investigating income-related outcomes in MS patients showed pronounced differences when compared to the general population. A previous Swedish study indicated a 28% difference in the proportions of people with MS and those without, receiving income from employment (39 and 67%, respectively) (24). Income is strongly linked to disability and serves as an indicator of the clinical progression of MS. One study reported that individuals with higher levels of physical disability were more likely to receive social benefits and less likely to have earnings (25). However, the lower cognitive function has been reported to affect income independently from a physical disability level, revealing the shortcomings of the EDSS in capturing the comprehensive picture of the patient (26).

Assessing income and employment outcomes can offer valuable insights into the effectiveness of treatment strategies. Data from the DMSR have demonstrated that early treatment can lower the risk of disability pension among patients with RRMS (27). Another DMSR study showed that a clinically stable disease course was associated with a decreased risk of losing income from salaries and a reduced risk of disability pension, emphasizing the importance of adequate treatment (28).

People with MS received more practical help and personal care assigned by the municipality than the matched individuals from the background population. This not only underlines the difference in accumulated disability in the two populations but also exemplifies why MS caretaking is associated with high economic costs, as also shown in previous studies (7, 8, 19, 20). A person's need for practical help and personal care can also impact close relatives. We found differences in family-related parameters among people with MS compared to controls. They were more likely to live alone, and a lower proportion was married at the reference date. Our results also confirmed previous findings that male participants with MS were more likely to be divorced compared to the background (29, 30).

Parenthood was also affected as a smaller proportion of people with MS had children; however, the difference in the number of children between parents with MS and without MS was negligible. One possible explanation is that the average age at first childbirth is lower than the average age at MS onset resulting in many individuals having established a family before receiving the MS diagnosis. Furthermore, the recommendations on family planning have undergone significant changes over the past few decades. In the past, women were advised not to have children, while contemporary women with MS are encouraged to pursue parenthood due to increasing possibilities for treatment and strong evidence that pregnancy does not interact adversely with the course of MS (31, 32).

This study has several limitations primarily due to its cross-sectional design. The cross-sectional design does not allow for the establishment of causal relationships between variables, and the obtained results may differ if the study were conducted at a different time (reference date) as this design does not take temporal changes into account. Consequently, we cannot project the future trajectory of the observed differences between people with MS and controls from the background population. Furthermore, all comparisons between the two groups are univariate and unadjusted for potential confounders, apart from the matching covariates.

When investigating working ability, the study lacked specific data on the nature of the participants' employment, which limits the ability to provide a more nuanced understanding of what contributes to differences in working ability. A previous Danish study showed that the likelihood of early pension for patients with physical work was 26% higher than that of patients with non-physical jobs (11).

Another limitation of the study is the generalizability of socioeconomic differences among individuals with MS as these differences are largely dependent on the comparability of social systems and societal structures across different countries. However, socioeconomic characteristics have been shown to be robust outcome measures, indicating that MS can have broad adverse consequences on various areas of life. When investigating the development of socioeconomic measures over a longer period, changes in social legislation should be taken into consideration which further complicates comparing results from different countries. Therefore, while the study provides valuable insights into the socioeconomic differences among individuals with MS, it is essential to consider the unique contexts of each country when interpreting the findings and comparing them to other studies conducted in different countries.

The strength of this large study lies in the completeness of data and the possibility to link Danish nationwide registries at the individual level. By matching on age, sex, ethnicity, and geographical region, the study was able to remove possible biases associated with these covariates. Adjusting for ethnicity is important as the background population in Denmark includes a significant proportion of 12% foreigners and descendants (compared to 0.8% in the Danish MS population), who may have different characteristics such as educational level, income, and family structure but also a different susceptibility for MS (33). Socioeconomic data, obtained from public registers, can capture other aspects of the disease such as fatigue and cognition, which physical disability measured by EDSS may not reflect. These data can serve as proxy parameters of disability or surrogate markers of the individual functional level.

In conclusion, MS can have a significant impact on the socioeconomical trajectory of an individual, which is particularly evident among elderly people with MS. This study highlights differences across multiple socio-economic domains such as education, employment, and family status. Therefore, when considering the comprehensive wellbeing of a patient, socioeconomic outcomes are important and robust measures of disability and individual function level. These measures reflect the broader consequences of MS on a person's life, extending beyond physical disability and providing a more holistic understanding of the challenges faced by individuals with MS.

## Data availability statement

The datasets presented in this article are not readily available because access to data is only available upon qualified request and approval by the Knowledge Center for Data Reviews (data responsible entity of the Capital Region of Denmark, approved by the Danish Data Protection Agency) and the Danish Multiple Sclerosis group. Requests to access the datasets should be directed at: www.dmsr.dk.

## Ethics statement

Ethical review and approval was not required for the study on human participants in accordance with the local legislation and institutional requirements. Written informed consent for participation was not required for this study in accordance with the national legislation and the institutional requirements.

## Author contributions

MW-H and MM conceived and designed the study. MW-H and RH wrote the first draft of the manuscript. MW-H, RH, and LP were responsible for statistical analyses. RH, MM, and FS did the review of concurrent medical literature. All authors assisted in the study design, were involved in the interpretation and final review of the data, drafting, or revising the manuscript for intellectual content, and approved the final version.
