# Supplementary material for: Socioeconomic status of the elderly MS population compared to the general population: a nationwide Danish matched cross-sectional study
Source: Front Neurol. 2023 Jun 13;14:1214897. doi: 10.3389/fneur.2023.1214897 (PMC10296197; doi:10.3389/fneur.2023.1214897)
Supplement: Supplementary file 1 [file Table_1.DOCX]

**Supplementary description 1: Level of education**

Two classification standards were used:

- International Standard Classification of Education (ISCED)
- Classification of education (DISCED)

We used the following ISCED levels to classify educational level:

- ISCED-level 0-2: Low Education
- ISCED-level 3-4: Medium Education
- ISCED-level 5+: High Education

**Chart of translation between Danish DISCED classification and English ISCED classification.**

| **ISCED-Level** | **Explanation** | **DISCED-Level** | **Danish Explanation** | **English Translation** |
| --- | --- | --- | --- | --- |
| 0 | Pre-Primary |  |  |  |
| 1 | Primary | 10 | Grundskole | Primary education |
| 2 | Lower Secondary | 15 | Forberedende kurser | Preparatory courses |
|  |  |  |  |  |
| 3 | Upper Secondary | 20 | Gymnasiale uddannelser | Upper Secondary Education |
| 4 | Post Secondary Non-Tertiary | 30 | Erhvervsfaglige uddannelser | Vocational Education and Training (VET) |
|  |  | 35 | Adgangsgivende uddannelsesforløb | Qualifying educational program |
|  |  |  |  |  |
| 5 | Short-cycle tertiary education | 40 | Korte videregående uddannelser, KVU | Short-cycle higher education |
|  |  | 50 | Mellemlange videregående uddannelser, MVU | Vocational bachelors educations |
| 6 | Bachelor’s or equivalent level | 60 | Bacheloruddannelser, BACH | Bachelors program |
| 7 | Master’s or equivalent level | 70 | Lange videreågende uddannelser, LVU | Masters program |
| 8 | Doctoral or equivalent level | 80 | Ph.d. og forskeruddannelser | Ph.D. program |

For a full explanation of the variable go to:

<https://www.dst.dk/da/Statistik/dokumentation/Times/moduldata-for-uddannelse-og-kultur/hfaudd>
