# Supplementary material for: Socioeconomic status of the elderly MS population compared to the general population: a nationwide Danish matched cross-sectional study
Source: Front Neurol. 2023 Jun 13;14:1214897. doi: 10.3389/fneur.2023.1214897 (PMC10296197; doi:10.3389/fneur.2023.1214897)

Supplementary Figure 1. Median annual income for the MS-population and the matched controls with an income above 0€ and not recipients of disability pension according to calendar year. Note that the income is adjusted for inflation by accounting for the net-price index. The rise in annual income displays the rise in purchasing power since 1980.

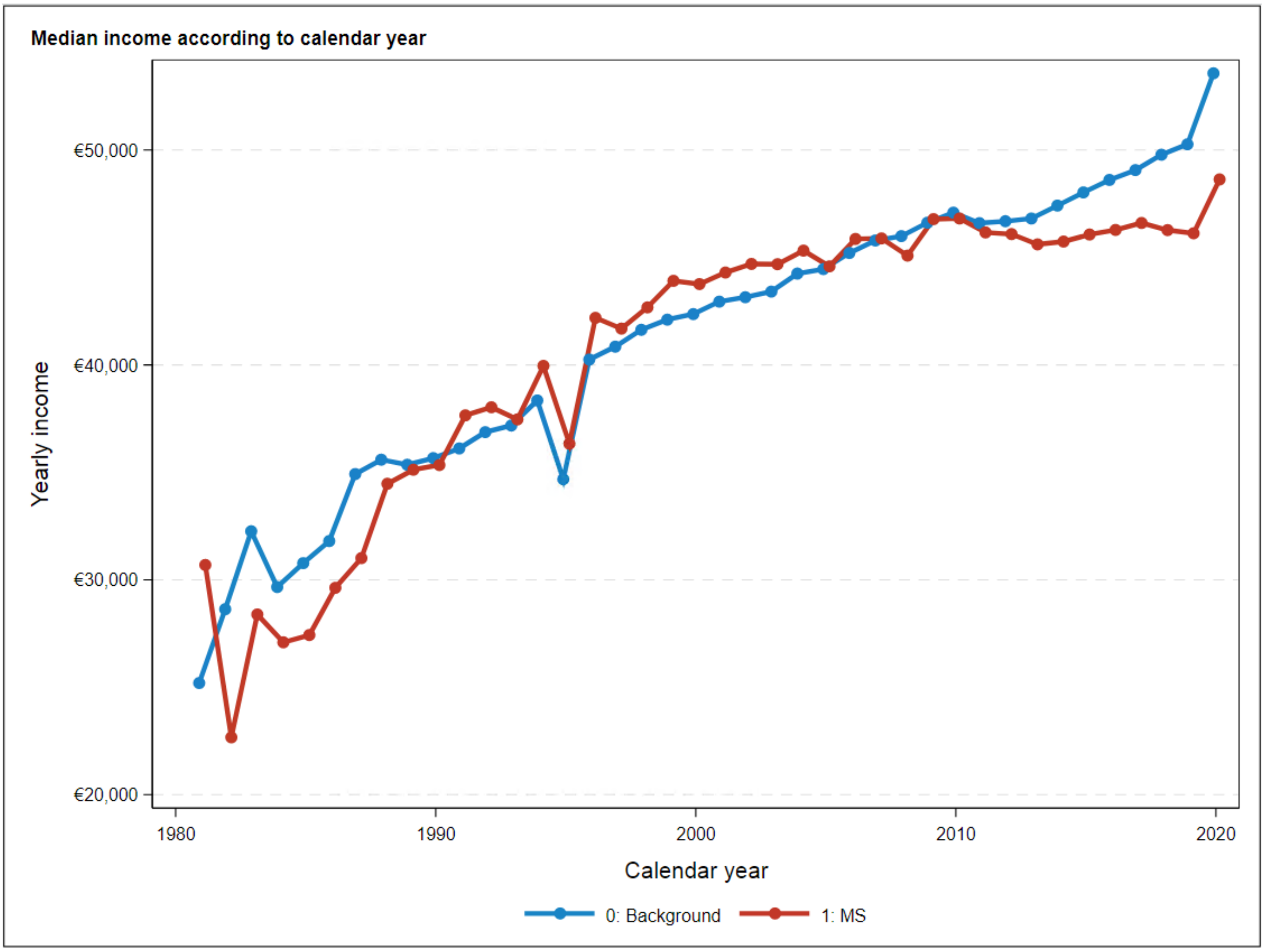

Supplement: Supplementary file 2 [file Data_Sheet_1.pdf]
